# Association between fat mass and obesity associated (FTO) gene rs9939609 A/T polymorphism and polycystic ovary syndrome: a systematic review and meta-analysis

**DOI:** 10.1186/s12881-017-0452-1

**Published:** 2017-08-21

**Authors:** Ai Ling Liu, Hui Jun Xie, Hong Yan Xie, Jun Liu, Jie Yin, Jin Song Hu, Cui Ying Peng

**Affiliations:** 1 Institute of Biological Science, School of Pharmaceutical and Biological Science, University of South China, Hengyang, Hunan Province 421001 China; 2 Hunan Province Cooperative Innovation Center for Molecular Target New Drug Study, Hengyang, 421001 China; 3 The Key Laboratory of Biological Toxicology and Ecological Restoration of Hengyang City, Hengyang, Hunan Province 421001 China

**Keywords:** Polycystic ovary syndrome, FTO, Meta-analysis, Polymorphism

## Abstract

**Background:**

Up to now, numerous case-control studies have reported the associations between fat mass and obesity associated (FTO) gene rs9939609 A/T polymorphism and polycystic ovary syndrome (PCOS), however, without a consistent result. Hence we performed current systematic review and meta-analysis to clarify the controversial results.

**Methods:**

Case-control studies reporting the relationship of rs9939609 A/T polymorphism and PCOS published before April 2015 were searched in Pubmed database without language restriction. Data was analyzed by Review Manager 5.2.

**Results:**

A total of five studies involving 5010 PCOS patients and 5300 controls were included for further meta-analysis. The results of meta-analysis showed that the FTO gene rs9939609 A/T polymorphism was significantly different between PCOS group and control group in different gene models (For AA + AT vs. TT: OR = 1.41, 95% CI = 1.28–1.55, *P* < 0.00001. For AA vs. AT + TT: OR = 1.54, 95% CI = 1.25–1.89, *P* < 0.0001. For AA vs. TT: OR = 1.74, 95% CI = 1.38–2.18, *P* < 0.00001. For A vs. T: OR = 1.36, 95% CI = 1.25–1.47, *P* < 0.00001, respectively) suggesting that A allele was a risk factor for PCOS susceptibility. Furthermore, subgroup analysis in Asian and Caucasian ethnicities also found significant association between rs9939609 A/T polymorphism and PCOS (In Asian subgroup: OR = 1.43, 95% CI = 1.29–1.59, *P* < 0.0001. In Caucasian subgroup: OR = 1.33, 95% CI = 1.08–1.64, *P* = 0.008)

**Conclusion:**

This meta-analysis suggests that rs9939609 A/T polymorphism of FTO gene is associated with PCOS risk, and that A allele is a risk factor for PCOS susceptibility simultaneously.

## Background

Polycystic ovary syndrome (PCOS), a common endocrine metabolic disorder in women of childbearing age, is characterized by polycystic ovary (PCO), oligomenorrhea or amenorrhea, clinical or biochemical hyperandrogenism, and insulin resistance. In addition to leading to infertility, PCOS is closely associated with diabetes mellitus type 2, cardiovascular diseases and endometrial carcinoma [1–3]. It was firstly reported in 1935 by Stein et al. [4], but its etiology still remains ambiguous. Existing data suggests that genetics is an important factor in the occurrence and development of PCOS [5, 6]. In recent years, a total of more than 100 candidate genes have been set out to explore the associations between single nucleotide polymorphism (SNP) and PCOS [7], such as fat mass and obesity associated (FTO) gene.

The human FTO gene is located on the chromosome 16q12.2 with a wide expression range of almost all tissues [8, 9]. The protein coded by FTO gene belongs to 2-oxoglutarate-dependent nucleic acid demethylase, involving energy metabolism [10]. In 2007, a genome-wide association study reported that FTO is associated with body mass index (BMI) and obesity [7]. Obesity is a common characteristic in PCOS patients. It is reported that more than 50% PCOS cases are overweight/obese [11]. So it is reasonable to assume that FTO gene might play a role in the pathogenesis of PCOS via BMI and/or obesity. Therefore, based on this assumption, many studies have been conducted to research the associations between FTO gene and the risk of PCOS.

Recently, a common single nucleotide polymorphism (SNP) (rs9939609) in the first intron of FTO gene with T to A change is widely investigated in PCOS. However, the results from different studies are controversial. Some studies observed the positive association of FTO with PCOS [12–15], while others studies found the opposite relationship [16–18]. A recent meta-analysis by Cai et al. showed that FTO rs9939609 polymorphism was associated with PCOS risk in East Asians but not in overall population [19]. Imperfectly, this meta-analysis considered only one genetic model and not all included studies were involved in FTO rs9939609 polymorphism. Therefore, we carried out current systemic review and meta-analysis to clarify the associations between the SNP rs9939609 and susceptibility to PCOS.

## Methods

### Search strategy

A comprehensive literature research was conducted in Pubmed to identify the studies aimed at exploring the association between FTO rs9939609 polymorphism and PCOS risk. The following keywords were used: “fat mass and obesity associated gene or FTO”, “rs9939609”, “polycystic ovary syndrome or PCOS”. All researched articles were updated on 30 March 2015 without any language limitation. When one eligible article was screened, we would check the references list of this paper manually to identify additional relevant publications.

### Selection of studies

Any study included in current meta-analysis must satisfy the following criteria: (a) Case-control studies aimed at investigating the association between FTO rs9939609 polymorphism and PCOS risk. (b) The number of genotypes and allele frequency in cases and controls were respectively available to calculate odds ratio (OR) with 95% confidence interval (CI). (c) Genotypes in control group meet the Hardy − Weinberg equilibrium (HWE) (d) PCOS patients accorded with one diagnose criteria of the following three: 1990 NIH, 2003 Rotterdam, and 2006 AE-PCOS Society [20–22].

### Data extraction and analysis

Two investigators (Liu and Xie) independently extracted data from the qualified articles. The following information were extracted: the name of first author, publication time, ethnicity, country, sample size, diagnose criteria in PCOS and controls, genotype distribution in both case and control groups, and *P* value of HWE in control group. If any disagreement was generated, another author would take part in to resolve it and make a final decision.

The pooled odd ratios (ORs) and 95% confidence intervals (CIs) were used to assess the strength of association between FTO rs9939609 polymorphism and PCOS. The associations were calculated under the following four genetic models: dominant model (AA + AT vs. TT), recessive model (AA vs. AT + TT), additive model (AA vs. TT) and allele model (A vs. T). Subgroup analysis classified by ethnicity was conducted to explore whether ethnicity has a special effect on this association.

Heterogeneity among studies was examined by Q-test and I^2^ statistic. When *P* < 0.05 for Q statistic or I^2^ > 50%, random effects model was used, otherwise fixed model was applied. To assess the stability of single study in overall effect, sensitivity analysis was performed by excluding each study once a time. Publication bias was estimated via funnel plot. All data analyses were performed by Review Manager 5.2. *P* < 0.05 was considered statistically significant.

## Results

### Characteristics of included studies

**Fig. 1:**
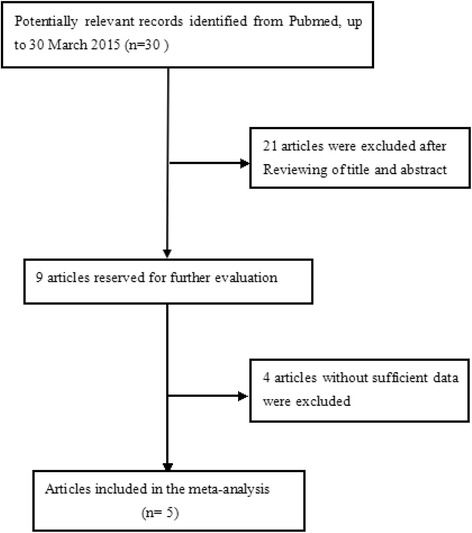
The flow chart of study selection

The flow diagram of study selection was presented in Fig. 1. Based on the inclusion criteria, a total of five studies exploring the association between FTO rs9939609 and PCOS were eligible for current meta-analysis, involving 5010 PCOS patients and 5300 controls [12–16]. All of the eligible studies provided the genotypes or allele information. One study was excluded because whether the genotypes distribution in PCOS was consistent with HWE was not mentioned, leading to the number of genotypes in PCOS group uncountable. The study by Li et al. [14] was a two-stage design (a GWAS and a replication study) with two populations. Because the two populations was the same ethnicity background (Han of Chinese), we considered them as one study. Among the five studies, two studies were conducted in Caucasian population and three studies were in Asian population. The diagnosis of PCOS in all studies was based on Rotterdam criteria. All the selected studies were consistent with HWE in controls. The main characteristics of the included studies are presented in Table 1.

**Table 1:** Characteristics of included studies in this meta-analysis

Authors	Year	Country/Ethincity	Definition		Sample size		PCOS			Control			HWE
			PCOS	Controls	PCOS	Control	AA	AT	TT	AA	AT	TT	
Barber et al.	2008	UK/Caucasian	Rotterdam criteria	Unknown	463	1336	99	231	133	212	644	480	0.870
Yan et al.	2009	China/Asian	Rotterdam criteria	Females proven fertile with normal menstrual cycle and ovarian morphology, and no family history of abnormal menses or hirsutism	215	227	5	55	155	1	43	183	0.361
Li et al.	2013	China/Asian	Rotterdam criteria	Regular menstrual cycle (26–35) and normal ovarian morphology	3599	3082	67	867	2665	29	563	2490	0.650
Kim et al.	2014	Korea/Asian	Rotterdam criteria	Women without special health problem	552	559	7	118	427	8	106	445	0.559
Ramos et al.	2015	Brazil/Caucasian	Rotterdam criteria	Non-hirsute,regular ovulatory cycles, no hormone drug taking at least 3 months before the study	181	96	39	83	59	17	47	32	0.971

*HWE* Hardy-Weinberg equilibrium

### Meta-analysis results

**Fig. 2:**
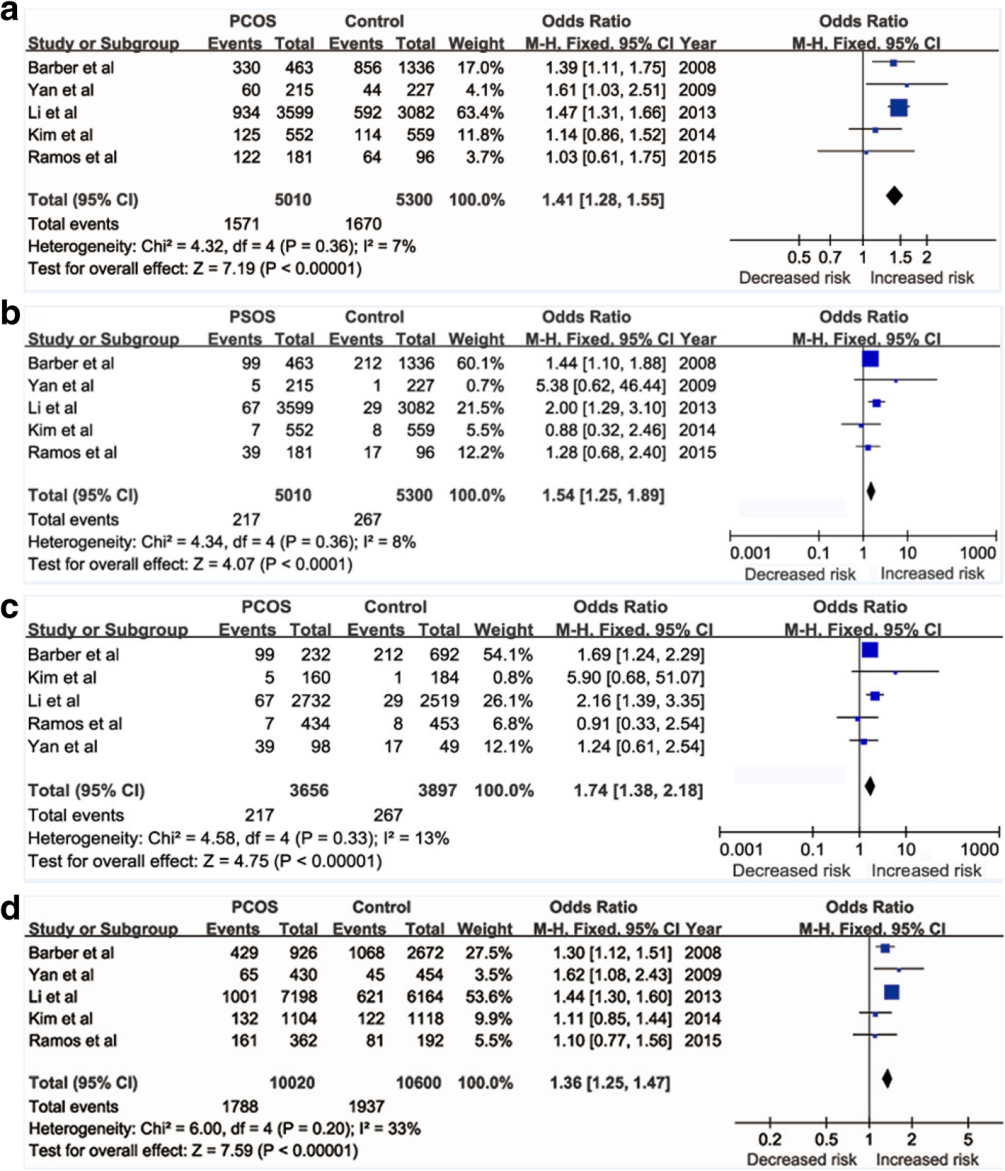
The association between FTO rs9939609 A/T polymorphism and risk of PCOS. (**a**) AA + AT vs. TT in overall analysis, (**b**) AA vs. AT + TT in overall analysis, (**c**) AA vs. TT in overall analysis, (**d**) A vs. T in overall analysis. For each study, the solid squares represent the ORs from the individual studies, horizontal lines represent corresponding 95% CIs. The pooled ORs and 95% CIs are shown by the shaded diamonds

The results of association between FTO polymorphism and PCOS risk were presented in Fig. 2. We found that the FTO rs9939609 A/T polymorphism was associated with PCOS risk under four genetic models (For AA + AT vs. TT: OR = 1.41, 95%CI = 1.28–1.55, *P* < 0.00001. For AA vs. AT + TT: OR = 1.54, 95% CI = 1.25–1.89, *P* < 0.0001. For AA vs. TT: OR = 1.74, 95% CI = 1.38–2.18, *P* < 0.00001. For A vs. T: OR = 1.36, 95% CI = 1.25–1.47, *P* < 0.00001, respectively), suggesting A allele was a risk factor for PCOS risk.

### Subgroup analysis

**Fig. 3:**
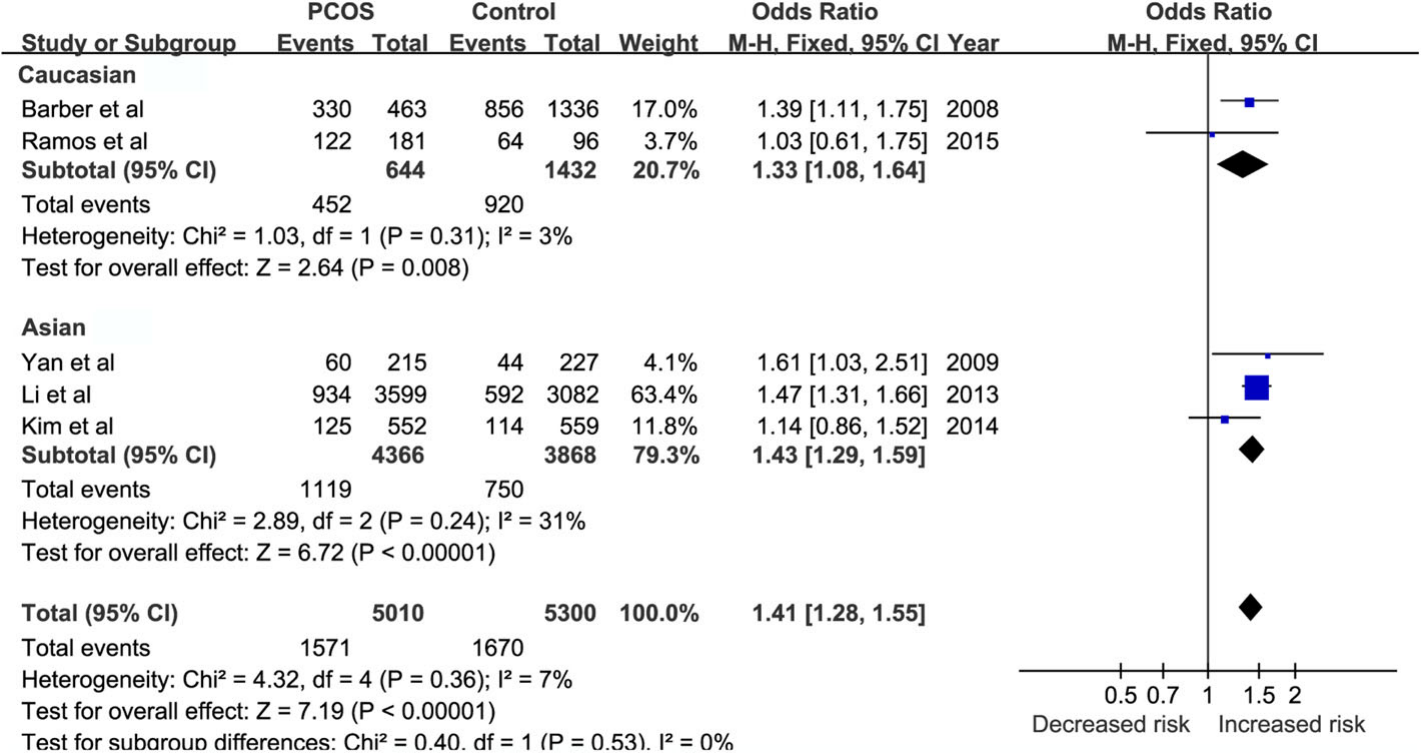
The association between FTO rs9939609 A/T polymorphism and risk of PCOS in subgroup model. The analysis was stratified by race. AA + AT vs. TT in subgroup (Caucasian and Asian) analysis of HWE > 0.05. The solid squares represent the ORs from the individual studies, horizontal lines represent corresponding 95% CIs. The pooled ORs and 95% CIs are presented as diamonds

In subgroup analysis stratified by ethnicity, significant association between FTO 9939609 A/T and PCOS risk was observed in Caucasian and Asian populations under dominant model (OR = 1.33, 95% CI = 1.08–1.64, *P* = 0.008; OR = 1.43, 95%CI = 1.29–1.59, *P* < 0.00001, respectively). The relative results were displayed in Fig. 3.

### Sensitivity analysis and publication bias

**Fig. 4:**
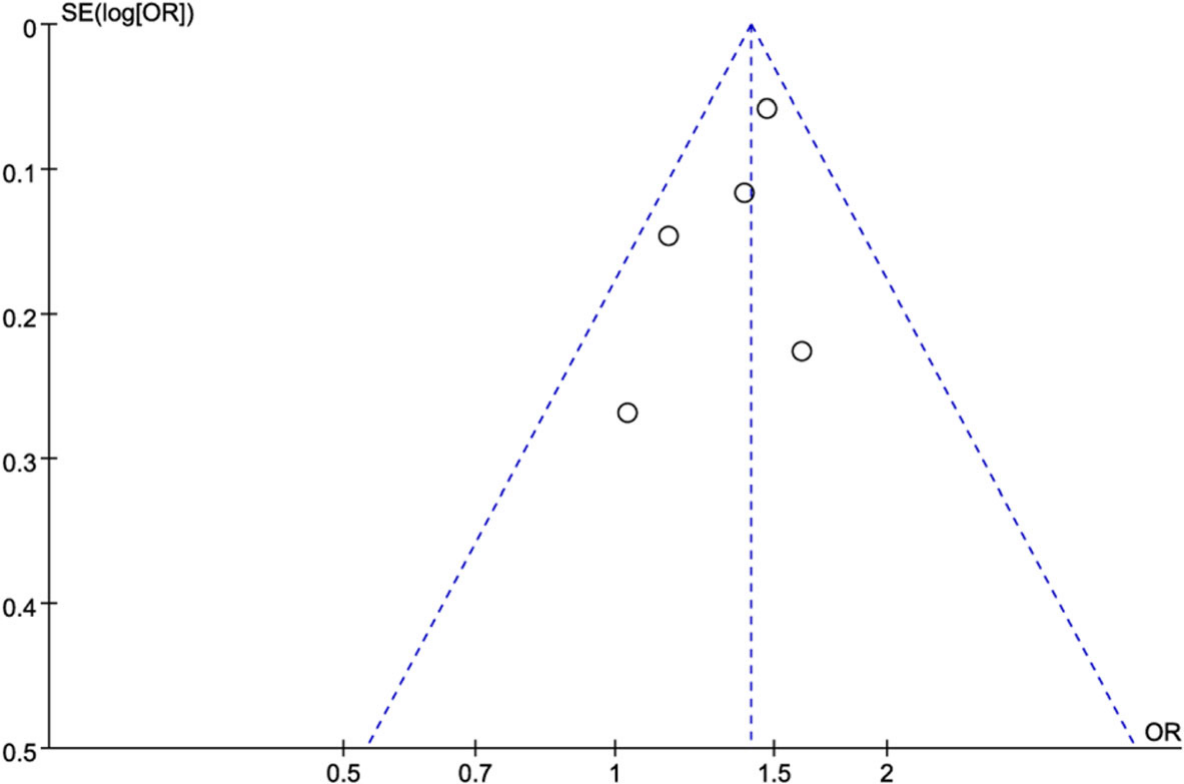
Funnel plot of publication bias in selection of studies on the FTO rs9939609 A/T polymorphism. Publication bias was assessed under dominant model (AA + AT vs. TT). SE represents standard error; OR represents odds ratio

To confirm the stability of above results, a sensitivity analysis was performed by removing studies sequentially. The results showed that the corresponding pooled ORs and *P* value under different genetic models were not changed significantly. Publication bias of the recruited studies was evaluated using funnel plot, the shapes of the funnel plots did not show significant asymmetry (Fig. 4).

## Discussion

As one of the most common endocrine disorder, the etiology of PCOS remains incompletely unknown. However, family studies have showed that genetic basis is responsible for PCOS and that no single gene can explain this syndrome entirely [23–25]. It is reported that 40–80% of PCOS women are overweight or obese [26–29]. Therefore, based on the high prevalence of obesity in PCOS and the heritable of obesity [30], genes associated with obesity might play a role in pathogenesis of PCOS such as FTO gene. Recent years, many studies have been conducted to identify the relationships between FTO rs9939609 and PCOS susceptibility. However, the results were inconsistent, making these associations more indistinct. The current meta-analysis was aimed at clarifying the association of FTO rs9939609 and PCOS.

In our meta-analysis, we found that FTO rs9939609 polymorphism was associated with PCOS under different genetic models. This finding is consistent with the study by Li et al. They performed a two stage study involving 3599 PCOS and 3082 control subjects in Chinese population [14]. They found that rs9939609 polymorphism of FTO is associated with PCOS in Chinese women, no matter the PCOS women are obese or non-obese. This indicated that FTO might interact with PCOS directly. However, because of insufficient data, this potential interaction way can not be re-analyzed in our meta-analysis. Contrary to our result, a case-control study by Kim et al. observed that FTO polymorphisms of rs9939609 are not major determinants of PCOS. But for women with PCOS, the variant allele of FTO rs9939609 was associated with increased BMI, suggesting FTO polymorphism contributes to PCOS risk through affecting BMI indirectly [16]. Therefore, the associations among PCOS, BMI/obese and FTO polymorphism still remain indistinct. FTO might indirectly play a role in PCOS risk via BMI/obese. Also, FTO might contribute to PCOS by directly interacting or via the combined direct and indirect ways. More studies are needed to demonstrate it.

In subgroup analysis, the significant association between FTO polymorphism and PCOS was found not only in Caucasian but also in Asian, suggesting ethnicity was not the potential source of heterogeneity. Partly in line with our result, a case-control study by Cai et al. observed that FTO rs9939609 polymorphism (or its proxy) was associated with PCOS only in East Asians, but not in Caucasians [19]. In Caucasians subgroup of study by Cai et al., one of the two included studies was about FTO rs9939609 and another was about FTO rs8050136. So, we inferred this point might the source of different conclusion compared with our subgroup result.

Several limitations of this meta-analysis should be acknowledged. First, the number of studies included in our meta-analysis was relatively small, thus leading to smaller studies in subgroup analysis and weaken statistical power. Second, potential sources of heterogeneity in this meta-analysis should include other factors such as BMI, waist-to-hip ratio (WHR), obese. However, because of limited data, we could not explore these factors in current meta-analysis. Finally, it is well known that the etiology of PCOS involving in gene-gene, and gene-environment interactions. However these interactions could not be investigated in our meta-analysis due to insufficient information.

## Conclusions

In conclusion, this meta-analysis provided evidence that the FTO rs9939609 polymorphism was significantly associated with risk of PCOS risk not only in Asians but also in Caucasians. Further large studies are needed to clarify the associations among the FTO rs9939609 polymorphism, BMI/obese and PCOS risk in the future.

This meta-analysis indicates that rs9939609 A/T polymorphism of FTO gene is associated with PCOS susceptibility, and that A allele is a risk factor for PCOS simultaneously.

## Abbreviations

BMI: Body mass index
CI: Confidence interval
FTO: Fat mass and obesity associated
HWE: Hardy − Weinberg equilibrium
OR: Odds ratio
PCO: Polycystic ovary
PCOS: Polycystic ovary syndrome
SNP: Single nucleotide polymorphism
WHR: Waist-to-hip ratio
